# Data sharing and the evolving role of statisticians

**DOI:** 10.1186/s12874-016-0172-9

**Published:** 2016-07-08

**Authors:** Nick Manamley, Steve Mallett, Matthew R. Sydes, Sally Hollis, Alison Scrimgeour, Hans Ulrich Burger, Hans-Joerg Urban

**Affiliations:** Amgen Ltd., 240 Cambridge Science Park, Cambridge, CB4 0WD UK; GlaxoSmithkline, Stockley Park West, 1-3 Ironbridge Road, Uxbridge, Middlesex UB11 1BT UK; MRC Clinical Trials Unit at UCL, Institute of Clinical Trials & Methodology, London, UK; MRC London Hub for Trials Methodology, Aviation House, 125 Kingsway, London, WC2B 6NH UK; AstraZeneca, Alderley Park, Cheshire, SK10 4TG UK; Centre for Biostatistics, Institute of Population Health, University of Manchester, Manchester Academic Health Science Centre, Oxford Road, Manchester, M13 9PL UK; Vectura, One Prospect West, Chippenham, Wiltshire SN14 6FH UK; Hoffman-La Roche, Grenzacherstrasse 124, Basel, 4070 Switzerland

**Keywords:** Statistician, Clinical trial, Data sharing, Transparency, Pharmaceutical research, Biotechnology research, Academic research

## Abstract

**Background:**

Greater transparency and, in particular, sharing of clinical study reports and patient level data for further research is an increasingly important topic for the pharmaceutical and biotechnology industry and other organisations who sponsor and conduct clinical research as well as academic researchers and patient advocacy groups. Statisticians are ambassadors for data sharing and are central to its success. They play an integral role in data sharing discussions within their companies and also externally helping to shape policy and processes while providing input into practical solutions to aid data sharing. Data sharing is generating changes in the required profile for statisticians in the pharmaceutical and biotechnology industry, as well as academic institutions and patient advocacy groups.

**Discussion:**

Successful statisticians need to possess many qualities required in today’s pharmaceutical environment such as collaboration, diplomacy, written and oral skills and an ability to be responsive; they are also knowledgeable when debating strategy and analytical techniques. However, increasing data transparency will require statisticians to evolve and learn new skills and behaviours during their career which may not have been an accepted part of the traditional role. Statisticians will move from being the gate-keepers of data to be data facilitators. To adapt successfully to this new environment, the role of the statistician is likely to be broader, including defining new responsibilities that lie beyond the boundaries of the traditional role. Statisticians should understand how data transparency can benefit them and the potential strategic advantage it can bring and be fully aware of the pharmaceutical and biotechnology industry commitments to data transparency and the policies within their company or research institute in addition to focusing on reviewing requests and provisioning data.

**Summary:**

Data transparency will evolve the role of statisticians within the pharmaceutical and biotechnology industry, academia and research bodies to a level which may not have been an accepted part of their traditional role or career. In the future, skills will be required to manage challenges arising from data sharing; statisticians will need strong scientific and statistical guiding principles for reanalysis and supplementary analyses based on researchers’ requests, have enhanced consultancy skills, in particular the ability to defend good statistical practice in the face of criticism and the ability to critique methods of analysis. Statisticians will also require expertise in data privacy regulations, data redaction and anonymisation and be able to assess the probability of re-identification, an ability to understand analyses conducted by researchers and recognise why such analyses may propose different results compared to the original analyses. Bringing these skills to the implementation of data sharing and interpretation of the results will help to maximise the value of shared data while guarding against misleading conclusions.

## Background

This article is one of a series of articles developed by the EFSPI (European Federation of Statisticians in the Pharmaceutical Industry) [1] and PSI (Statisticians in the Pharmaceutical Industry) [2] Data Sharing Working Group. The Working Group consists of medical research statisticians from the pharmaceutical and biotechnology industries and academia, with the intention of providing knowledge and insights regarding the practical challenges and opportunities of accessing research data for re-analysis or secondary research purposes.

The intended audience for this article comprises parties interested in the value statisticians can add to the topic of data sharing within the pharmaceutical or biotechnology industries and academic research groups. Also for statisticians whose role may evolve due to data transparency, this paper intends to highlight areas and skills statisticians may not have considered as part of their role.

Prior to the introduction of EMA policy 0070 [3] on data sharing, a statistician’s role in accessing external data would have been limited to gleaning information on other companies’ results from published manuscripts or from websites such as www.clinicaltrials.gov. Following the introduction of the EMA policy 0070 the subject has become embedded in the culture of large pharmaceutical and biotechnology companies, and longstanding academic trials units; although, perhaps due to resource constraints or fewer opportunities, to a lesser extent in some small companies and certain academic research groups. However, this is a continually evolving area. For many years, large pharmaceutical and biotechnology companies have had policies in place regarding access to Clinical Study Reports (CSRs); the EMA draft policy 0070 on data sharing prompted the industry into creating further policies to address the sharing of individual patient level data (IPD) from clinical trials they conducted. Whilst EMA policy 0070 makes reference to sharing of IPD, there is currently no regulatory mandate to do so. The majority of pharmaceutical and biotechnology companies have responded positively to this call for increased transparency believing it’s a positive step for industry, researchers and the public. In 2014, the PhRMA/EFPIA (Pharmaceutical Research and Manufacturers of America, European Federation of Pharmaceutical Industries and Associations) ‘Principles for Responsible Clinical Trial Sharing’ stated a commitment to enhancing public health through responsible sharing of clinical trial data in a manner that is consistent with principles to safeguard the privacy of patients, respecting the integrity of national regulatory systems, and maintaining incentives for investment in biomedical research. Implementation started in January 2015 of five specific commitments to enhance data sharing with researchers, enhance public access to clinical study information, share results with patients who participate in clinical trials, certify procedures for sharing clinical trial information, and reaffirmation of commitments to publish clinical trial results. Data sharing has also provoked an unprecedented collaboration between companies with such initiatives as the Multi-User Clinical Study Data Request (CSDR) Platform (www.clinicalstudydatarequest.com) which continues to develop and increase in subscribers. The platform was developed by large pharmaceutical companies (initiated by GlaxoSmithkline) with the view that this could become a viable platform for all data holders, i.e., large, medium and small companies and, potentially, academic institutions. To date, only pharmaceutical and biotechnology companies have signed up, however, there are other models under discussion with institutes such as the Multi-Region Clinical Trials (MRCT) Center of Harvard and Brigham and Women’s Hospital who advocate a centralised single-platform with the ability to upload and download data with the capability to grow and add data from new sponsors [4].

Statisticians are ambassadors for data sharing and are central to its success. They play an integral role in data sharing discussions within their respective companies and also externally helping to shape policy and processes while providing input into practical solutions to aid data sharing. Secondary use of data is implicit in public-sector trials, and access to data is aligned with the values of many pharmaceutical and biotechnology companies related to benefits to society and patients; some companies and individuals remain cautious regarding the benefits and sceptical of how the data will be used in particular where there is the potential to make unfounded health scares involving their assets.

Traditionally, the roles and responsibilities of a statistician have tended to be focussed on providing strategic input to pre-clinical research, clinical development plans, designing clinical trials, generating clinical evidence to support regulatory submissions, reimbursement submissions (payers) and publications as well as supporting post-marketing activities [5]. In general, discussions with external parties regarding analysis methods and inferences have tended to focus on formal requests from regulatory agencies and reimbursement agencies, and responding to peer review comments from submitted manuscripts. In contrast, statisticians in academic institutions have focussed on publications with a strong desire to access data retained by pharmaceutical and biotechnology companies. To date, for the majority of statisticians, there has been little time spent collaborating between pharmaceutical and biotechnology companies and other (e.g. academic) research organizations in terms of data sharing. However with a future trend towards greater transparency, building public trust and increased data sharing, engagement between these stakeholders will increase.

Statisticians possess many qualities such as negotiation, collaboration, diplomacy, written and oral skills and an ability to be responsive and knowledgeable when debating analytical techniques; however data transparency will require the majority of statisticians from industry to learn new skills and ways of working. If not already engaged with external activities, statisticians within the pharmaceutical and biotechnology industry and academia will increasingly expand their role to become more embedded in the data facilitation process. To adapt successfully to this new environment, statistical leaders within the pharmaceutical and biotechnology industries and academia will work towards broadening the role of the statistician, and define new responsibilities that lie beyond the normal boundaries. Building trust with the public through greater transparency and increased engagement with academic researchers will bring many benefits, but these will inevitably come at a cost in terms of time and resources, which will be particularly acute for smaller companies, and will require commitment from industry leaders to address these demands.

## Discussion

### Overview on anticipated changes

Data sharing is generating changes to the traditional role of a statistician within the pharmaceutical and biotechnology industry, as well as academic institutions. This will affect a number of areas of their work including: 1) An understanding of what data transparency can offer statisticians, 2) An increased ability to rapidly understand other study designs and analytical techniques, 3) More thorough documentation of analysis methods and data manipulation, 4) An increased focus on being able to utilize opportunities of data sharing to benefit their own work and the ability to understand and replicate analyses conducted by other researchers, 5) An increased focus on consultancy skills and a collaborative attitude to manage an external network, and 6) Expertise in re-analysing data with modern analytical techniques.

Opportunities for how the role of statisticians may evolve with increased sharing of clinical trial data are discussed.

### What can data transparency offer statisticians?

As well as focusing on requests for data, statisticians should understand how data transparency can benefit them and the potential strategic advantage it can bring, be fully aware of the pharmaceutical and biotechnology industry commitments to data transparency, and the policies within their company or research institute. Since discussion started in relation to data transparency the focus has been on academic researchers requesting data from pharmaceutical or biotechnology companies; but what of the reverse scenario where pharmaceutical/biotechnology industry statisticians request data from academic researchers or other pharmaceutical/biotechnology companies? Although this is possible, to date we are not aware of a company requesting IPD generated through a clinical trial conducted by another company. Some have requested CSRs as these company policies have been in place for a number of years. However, we consider this to be a matter of time where in the future the sharing of data between companies will be the norm alongside the increasing number of research collaborations. Thus, statisticians in the pharmaceutical industry may become exposed to an academic-like working style, with regard to aspects such as the interpretation of data from other researchers. This may be a further opportunity for statisticians to be valued as strategic thinkers. Access to additional external data will have advantages aiding the design of studies. For example, it will help to understand patterns of missing data, predicting drop-outs, provide good prior distributions and aid in predicting study success, etc.

### Study design and implementation

Pharmaceutical and biotechnology companies might be inclined to align on optimal study designs and the correct data to be collecting. Linking in with core datasets initiatives such as COMET (Core Outcome Measures in Effectiveness Trials; www.comet-initiative.org) will become important for statisticians. The COMET Initiative brings together people interested in the development and application of agreed standardised sets of outcomes, known as ‘core outcome sets’. These sets represent the minimum that should be measured and reported in all clinical trials of a specific condition, and are also suitable for use in clinical audit or research other than randomised trials. There is an expectation that the core outcomes will be collected and reported, making it easier for the results of trials to be compared, contrasted and combined as appropriate.

### Thorough documentation of analysis methods and data manipulation

Key to the success of data sharing will be thorough and accurate documentation of analysis methods and data manipulation techniques. A statistician produces an analysis plan documenting the analysis techniques and also documents any data manipulations required to convert data from that collected and entered into the database into data which is analytically useable; the only industry standards in place to do this are from the Clinical Data Interchange Standards Consortium (CDISC) for converting raw data to the Study Data Tabulation Model (SDTM); SDTM is a documented standard in the Pharmaceutical Industry for converting raw data into a standardised format which should be more analytically useable though may require more effort for the user to understand. The quality of documentation produced within the industry varies considerably, particularly relating to the conversion from SDTM to Analysis Data Model (ADaM) datasets; ADaM datasets are considered analysis-ready by the pharmaceutical industry. It would be naïve to suggest a statistician, without knowledge of a company or research group’s processes, or knowledge of CDISC could reliably reproduce the analysis of a clinical trial. Generally, the pharmaceutical/biotechnology industry will document data manipulations using statistical programming code (e.g. SAS, R) in a spreadsheet or database, but this will mean very little to a researcher unfamiliar with the statistical programming language used. In academic and small pharmaceutical or biotechnology companies, the level of documentation is likely to be less and data manipulation unlikely to be using CDISC standards for SDTM due to knowledge and resources. In fact, knowledge of SDTM and terminology may not exist in these settings. Clearly the data requestor will need to understand the data and statistical methodology before undertaking their research and the data holder should thoroughly document this. Communication and clear documentation will be needed between data holders and researchers along with further collaboration and more industry standards and best practices. In some cases a company or research institute, may use a Contract Research Organisation (CRO) which introduces a third party into the communication chain; a thorough understanding of roles and responsibilities will need to be further assessed under these circumstances.

Irrespective of the problems related to documenting data manipulation, there are additional questions to consider. Can an analysis plan be followed accurately enough to reproduce an analysis? Is a CSR the correct place to document deviations from an analysis plan? Furthermore, how are analyses potentially un-documented in the CSR, e.g., additional analyses required for manuscripts, reimbursement dossiers, etc. to be documented? The statistician generating these analyses may not be associated with the company or research institute when the data request is received and therefore it is critical to document analytical decisions at the point of its conception and/or completion. Understandably there will always be some scepticism of how the data has been manipulated and whether the data has been deployed in such a way as to bias the results towards the primary objectives and alternative hypotheses. But equally, statisticians are also inclined by training towards accepting the null hypothesis to be “true” unless there is significant evidence to the contrary. Additionally statistician’s training is to reduce bias without compromise and all pharmaceutical/ biotechnology statisticians work in a heavily regulated environment where any analytical technique or data manipulation is open to scrutiny by international and pan-European agencies. Therefore, data transparency should reduce the external scepticism as researchers become more confident in the quality of industry’s analyses and data manipulation processes; we are seeing this to a certain extent already. For example, In 2005 JAMA adopted a policy that included a requirement for independent statistical analysis by an academic biostatistician for industry-sponsored and industry-analysed studies due to several high-profile trials that had evidence of problems with data integrity, inappropriately conducted statistical analyses, and incomplete reporting of major findings. In 2013, JAMA changed their policy to consider, for publication, clinical trials that are analysed by statisticians employed by or contracted by the study sponsor, without requiring independent statistical analysis by an academic biostatistician. JAMA found that over the previous two years, the conduct of additional analyses by independent academic biostatisticians generally did not result in meaningful changes in the study results [6].

### Increased technical skills

External collaboration will necessitate statisticians to have the ability to understand and replicate analyses conducted by other researchers. Although there are many statisticians already undertaking such research and discussions, an increased number of statisticians will be required to have expertise in reviewing and interpreting systematic reviews and meta-analyses, to conduct evaluations of methods used, and to provide a response where methods may be flawed or lead to false conclusions. An open-minded approach is needed requiring research into different statistical methods in order to fully understand the research that has been conducted.

Statistical methods continually evolve with innovation or in reaction to specific challenges, sometimes made possible only with modern computational power, and a researcher may want to conduct an investigation using a more powerful method than that used in the primary analysis. If the conclusions differ, the reaction from the data-holder may be to defend the original analysis. Rather than a culture of defending the original analysis as being correct, and fearing that an alternative analysis may give a different conclusion, statisticians should foster a spirit of openness and curiosity particularly where methods are potentially more advanced. If the conclusions are not robust, in terms of different analysis methods, then statisticians should examine what assumptions are being made, and explore any inconsistencies.

### Data management expertise

Statisticians have played major roles in defining IPD systems and process development such as CSDR while also shaping policies related to access to clinical trial data. Statisticians can leverage their experience as data access experts with regard to data sharing both internal and external to their employer. External data sharing is about providing access to data but also provides a support mechanism around data structure and advice on potential analyses. Openness and a strong collaborative attitude are significant attributes required for data sharing to be successful in order to minimise the risk of publication of misleading results.

### Data redaction and anonymisation

Technical advances will not only be statistical. Statisticians will probably be the primary contact for data redaction and anonymisation requiring significant knowledge of data privacy legislation along with relevant guidance documents [7–15]. To understand the data, a researcher may attempt to reproduce the primary analysis of a study. This can prove difficult in certain situations depending on the anonymisation technique. For example, one potential technique for maintaining anonymisation is to move dates by a random number of days or replace dates with days from randomization or first dose. Reproducing analyses in these situations is generally easily facilitated but that is not the case in situations where diseases are seasonal, e.g., asthma or allergies such as hay fever. Furthermore, informed consent considerations may lead to all patients from selected countries being removed prior to data being shared. Statisticians will need technical knowledge to understand the analysis methods balanced with data privacy requirements to minimise the probability of re-identification. In terms of re-identification of redacted data in a CSR and IPD, in the future there is likely to be an expectation that a company statistician makes an estimation of the probability of an individual being re-identified.

### Consultancy skills

When the data sharing models (e.g. CSDR, Yale University Open Data Access [YODA; www.yoda.yale.edu]) were put in place it was a deliberate requirement by the pharmaceutical and biotechnology industries to maintain the independence of the data-requestor with no influence from the data sharer in receiving, reviewing, or approving data sharing requests. However, some data sharing models have been set up to allow collaboration between the data holder and researchers requesting access to data. Independence has been maintained, although it has been possible for some contact between the researcher and data-holder to respond to questions related to the data and analytical technique. However, there is the potential for data sharing to be more collaborative. For this to be successful, core competencies for statisticians will include strong written and oral communication skills, which although accepted as a requirement now, will need to be of an alternative style when collaborating with external researchers with a low emphasis on individual company communication styles and a higher importance on language understandable between multiple communities. In addition, collaboration skills, especially with academia, and an ability to react to different opinions will be required. This includes an ability to be responsive to requests, to listen and learn, and to supplement strong analytical skills with the ability to engage openly with external researchers. Academic researchers may be seeking answers to different questions compared to regulators and payers, and researchers may have very different objectives compared to those of the clinical development plan for a product. For example, researchers may be more concerned with defining clinically-relevant outcomes at the individual patient level, rather than looking at population-based averages which are often the focus of clinical research supporting regulatory submissions. Where differences of opinions do occur it is important to follow good scientific and statistical principles, and be mindful that the ultimate focus of all clinical research should be on patients.

As CSRs become widely available, a more intensive review and critique of CSRs will bring more questions from research and patient groups. As a reaction, companies will be more careful in the interpretation of results within the CSR and reduce interpretations to the minimum demanded by the objectives of the study. This may make CSRs also increasingly straight forward to interpret from a statistical point of view. Requests from researchers to sponsors have primarily focused on data provision, but in the future more collaboration on additional analyses beyond the CSR content will be needed rather than a request for the IPD itself. Being able to discuss questions posed by researchers will require statistical support and the statistics departments should be prepared for this.

## Summary

Data transparency will lead to some changes within the pharmaceutical and biotechnology industry as well as academia and other research bodies. The majority of those parties are overwhelmingly supportive and believe future research will build trust between associations and will be of benefit to patients. The typical role of all statisticians within the pharmaceutical and biotechnology industries, academia and research bodies will require adaptations over time.

Key areas of expertise required by statisticians currently include: Strong collaborative skills, good written and oral communication skills, an ability to listen and be responsive to requests from colleagues and expertise in explaining the appropriateness of statistical methods of analysis.

In the future additional skills will be required to manage opportunities arising from data sharing to a level which may not have been an accepted part of their traditional role or career. Statisticians will need to be fully aware of the pharmaceutical and biotechnology industry commitments to data transparency and the policies within their company or research institute and have strong scientific and statistical technical skills applicable to supplementary analyses as part of any collaboration with other researchers. They will also require enhanced consultancy skills, in particular the ability to defend good statistical practice in the face of criticism and the ability to critique methods of analysis. While also having expertise in data privacy regulations, data redaction and anonymisation and be able to assess the probability of re-identification, statisticians will also need an ability to understand analyses conducted by researchers and recognise why such analyses may propose different results compared to the original. Bringing these skills to the implementation of data sharing and interpretation of the results will help to maximise the value of shared data while guarding against misleading conclusions.

## Abbreviations

ADaM, analysis data model; CDISC, clinical data interchange standards consortium; COMET, core outcome measures in effectiveness trials; CRO, contract research organization; CSDR, clinical study data request; CSR, clinical study report; EFPIA, European Federation of Pharmaceutical Industries and Associations; EFSPI, European Federation of Statisticians in the Pharmaceutical Industry; EMA, European Medicines Agency; FDA, Food and Drug Administration; ICH, International Council for Harmonisation; IPD, individual patient data; ITT, Intent-to-Treat; JAMA, Journal of the American Medical Association; MA, meta-analysis; MRCT, multi-region clinical trials; NMA, network meta-analysis; PhRMA, Pharmaceutical Research and Manufacturers of America; PSI, Statisticians in the Pharmaceutical Industry; SAP, statistical analysis plan; SDTM, study data tabulation model; STRATOS, strengthening analytical thinking for observational studies; YODA, Yale University Open Data Access.
